# The success of biomaterial-based tissue engineering strategies for peripheral nerve regeneration

**DOI:** 10.3389/fbioe.2022.1039777

**Published:** 2022-10-18

**Authors:** Yuhui Jiang, Xiaoxuan Tang, Tao Li, Jue Ling, Yumin Yang

**Affiliations:** ^1^ Medical School of Nantong University, Nantong University, Nantong, China; ^2^ Key Laboratory of Neuroregeneration, Ministry of Education and Jiangsu Province, Co-innovation Center of Neuroregeneration, Jiangsu Clinical Medicine Center of Tissue Engineering and Nerve Injury Repair, Nantong University, Nantong, China

**Keywords:** nerve regeneration, locomotor disorder, biomaterials, neural stem cells, bionic grafts

## Abstract

Peripheral nerve injury is a clinically common injury that causes sensory dysfunction and locomotor system degeneration, which seriously affects the quality of the patients’ daily life. Long gapped defects in large nerve are difficult to repair *via* surgery and limited donor source of autologous nerve greatly challenges the successful nerve repair by transplantation. Significantly, remarkable progress has been made in repairing the peripheral nerve injury using artificial nerve grafts and a variety of products for peripheral nerve repair have emerged been approved globally in recent years. The raw materials of these commercial products includes natural/synthetic polymers, extracellular matrix. Despite a lot of effort, the desirable functional recovery still remains great challenges in long gapped nerve defects. Thus this review discusses the recent development of tissue engineering products for peripheral nerve repair and the design of bionic grafts improving the local microenvironment for accelerating nerve regeneration against locomotor disorder, which may provide potential strategies for the repair of long gaps or thick nerve defects by multifunctional biomaterials.

## 1 Introduction

Peripheral nerve injury mainly occurs in severe accidents such as work-related injuries and traffic accidents often accompanied by symptoms such as sensory and locomotor dysfunction, and it is poor repair would seriously affect the quality of the patients’ daily life (Yi et al., 2019; Vijayavenkataraman, 2020). Peripheral nerve injury can be divided into traction injury, cutting injury, firearm injury, ischemic injury and iatrogenic injury. The phenotypes of peripheral nerve injury include nerve entrapment, nerve hemisection, nerve transection, and nerve defects (Spinner and Kline, 2000; Tezcan, 2017). Significantly, peripheral nerve injury can result in varying degrees of neuronal degeneration, muscle atrophy, and fibrosis (Yang et al., 2022c). Due to the slow rate of regeneration (approximately 1 mm/day), irreversible atrophy usually occurs before skeletal muscle reinnervation. This seriously affects the functional reconstruction of target muscles and the recovery of locomotor function after nerve injury (Sun et al., 2021). Therefore, assessment of functional recovery is critical to determine the degree of regeneration following peripheral nerve injury (Navarro, 2016), which is especially true for studies devoted to developing tissue-engineered products to replace autologous nerve grafts for peripheral nerve injury. (Angius et al., 2012; Heinzel et al., 2020).

Traditionally, autologous nerve repair is the golden line for repairing the injured peripheral nerves. However, it still has many disadvantages: 1) Repairing the defective nerve with normal nerves from other parts may cause secondary surgical damage; 2) cable-like repair is not accurate 3) It is easy to cause mismatched growth of regenerative nerves, resulting in neuroma; 4) Limited source can hardly meet the needs of long gaps or thick nerve defects, and therefore limited its clinical applications (Rutkowski and Health, 1998; Gvans, 2000).

Thanks to the continuous development of biomedical materials for nerve repair, remarkable progress has been made in the application of artificial nerve grafts in repairing the peripheral nerve injury (Gu et al., 2011; Gu et al., 2015a). There have been a variety of products for peripheral nerve repair emerged in recent years. Generally, the ideal tissue-engineered nerve grafts should be compounded with trophic factors and bionic structures within the bioscaffold (Tang et al., 2021b; Zhang Y. et al., 2022; Chen et al., 2022). Furthermore, various kinds of cells can be seeded into bioactive implants to promote the recovery of nerve function by differentiating into locomotor neurons to innervate muscles or secreting neurotrophic factors to repair injury (Du et al., 2018). Therefore, how to choose ideal seed cells and neurotrophic factors with appropriate concentration and combination to further equip tissue engineering products for peripheral nerve repair with better biosafety and bioactivity for functional reconstruction of nerve tissue is a crucial in this fields. Thus, this review discusses the research status and latest progress of tissue engineering products for peripheral nerve repair.

## 2 Current approved nerve repair grafts in China

Peripheral nerve repair grafts that have been approved in China belong to Class III medical devices. Until March of 2022, the Chinese Food and Drug Administration has approved a total of five domestic and two imported medical device products (Table 1). The raw materials of these products include collagen, chitosan, polyglycolide lactide (PGLA), DL-lactide-co-ε-caprolactone and polyglycolic acid (PGA), extracellular matrix (ECM).

**TABLE 1:** Summary of commercially available peripheral nerve repair grafts.

Name	Producer	Main ingredients	Year of registration (NMPA registration certificate No.)	Application
Decellular allograft nerve repair material (Shenqiao)	Guangzhou Zhongda Medical Equipment Co., Ltd.	Collagen fibers ECM	2012 (20163461598)	1–5 cm traumatic sensory nerve defects
Acellular Matrix Peripheral Nerve Repair Membrane	Shandong Junxiu Biotechnology Co., Ltd.	ECM of peripheral nerve (derived from porcine)	2019 (20193130355)	Peripheral nerve injury without parenchymal defects or transanastomosis
Artificial nerve sheath tube	Beijing Tianxinfu Medical Equipment Co., Ltd.	Collagen type I (from bovine Achilles tendon)	2016 (20163462399)	Peripheral nerve defect (≤ 2 cm)
Peripheral Nerve guide	Beijing Huifukang Medical Technology Co., Ltd.	Chitosan	2021 (20213130298)	Non-pathological upper limb median nerve, ulnar nerve and radial nerve disconnection injury used for non-pathological nerve injury (≤ 2 cm)
Peripheral nerve repair graft	Jiangsu Yitong Biotechnology Co., Ltd.	Chitosan、Chitin、Gelatin、PGLA	2020 (20203130898)	Sensory nerve function repair of finger nerve, superficial radial nerve branch and median forearm nerve defects within 3 cm
Neurolac Peripheral Nerve guide	Polyganics Innovations B.V.	DL-lactide-co-ε-caprolactone	2016 (20163130201)	Peripheral nerve defect (≤ 2 cm)
GEM Neurotube	Synovis Micro Companies Alliance, Inc	PGA	2017 (20173463001)	0.8–1 cm nerve defects to restore sensory function

The decellularized allogeneic nerve repair material (trade name: Shenqiao) is produced by Guangzhou Zhongda Medical Instrument Co., Ltd., which is made from the peripheral nerves collected from the human body (cadavers) followed by decellularization. It is mainly composed of collagen fibers and the extracellular matrix retaining the structure of nerve basement membrane tube, perineurium, epineurium and other supporting structures, which is suitable for repairing 1–5 cm traumatic sensory nerve defects.

Acellular matrix peripheral nerve repair membrane produced by Shandong Junxiu Biotechnology Co., Ltd. Has obtained the National Medical Device Registration Certificate in 2019. It is a transparent or translucent film based on the extracellular matrix of porcine peripheral nerves, which retains the nerve scaffold structure and the active ingredients of the abundant nerve extracellular matrix. This membrane is suitable for auxiliary repair of non-defective or anastomosed peripheral nerve injury.

Artificial nerve sheath tube produced by Beijing Tianxinfu Medical Equipment Co., Ltd. is suitable for repairing peripheral nerve defects those are less than 2 cm obtained the National Medical Device Registration Certificate in 2016. The material of this product is mainly derived from the bovine achilles tendon with high-purity type I collagen and made into a spongy collagen sheath to guide nerve growth. It has good biocompatibility and can be degraded and absorbed within 3–6 months, degradation products of which are amino acids and water.

Peripheral nerve repair graft from Jiangsu Yitong Biotechnology Co., Ltd. is composed of a catheter and a built-in fiber to guide directed nerve growth. The catheter is prepared by freeze-drying chitosan, chitin and medicinal gelatin. The built-in fiber is polyglycolide lactide (PGLA) fiber. The graft is developed for repair of sensory function in the digital nerve, superficial branch of the radial nerve, and median nerve of the forearm with a defective length of less than 30 mm, which can be degraded and absorbed within 3 months.

Neurolac Peripheral Nerve guide and GEM Neurotube are two imported products from Polyganics Innovations B.V. in Netherlands and Synovis Micro Companies Alliance, Inc., which were both awarded the National Imported Medical Device Registration Certificate. Neurolac Peripheral Nerve guide possesses a tubular structure to repair peripheral nerve defects up to 20 mm, prepared from (DL-lactide-co-ε-caprolactone) copolymer. The absorbable GEM Neurotube is an polyglycolic acid mesh tube, of which the pipe wall is corrugated to increase its strength and flexibility. It can be absorbed into the human body through a hydrolysis process and used to repair 8–30 mm digital nerve defects to restore the sensory function.

## 3 Research progress of peripheral nerve grafts

Although a variety of tissue engineering products for peripheral nerve repair have been approved, several disadvantages still exist, such as the mismatch between material degradation and nerve regeneration, the inability to repair long-distance injuries, poor locomotor functional recovery (Gu et al., 2014; Wu et al., 2017). In recent years, many studies have focused on selection of raw materials, construction of biomimetic structures, improvement of the regenerative microenvironment and implantation with seed cells.

### 3.1 Raw materials

Ideal biomaterials should have good biocompatibility, biodegradability and minimal immunogenicity, be non-toxicity, non-teratogenic and non-carcinogenic, supporting for nerve regeneration and recovery, etc. (Nectow et al., 2012; Fakhri et al., 2020). The chemical and physical properties of biomaterials directly affect their therapeutic effect for nerve regeneration, emphasizing the importance of materials selection for tissue-engineered nerve grafts. Recently, many biodegradable materials have been used in designing scaffolds for neural tissue engineering, including synthetic polymers such as polyglycolic acid (PGA) (Kusuhara et al., 2019; Sayanagi et al., 2020), polylactic acid (PLA) (Jahromi et al., 2020), polyglycolide lactide (PGLA) (Xu et al., 2017; Dos Santos et al., 2019; Lu P. et al., 2021; Manto et al., 2021), poly (ε-caprolactone) (PCL) (Dong et al., 2020; Dursun Usal et al., 2022) and polyurethane (PU) (Yang H. et al., 2022), etc. For example, Reid et al. used a thin film of PCL to prepare nerve catheter for repairing 10 mm sciatic nerve injury in rats, and after 18 weeks, the growth rate of regenerated axons in PCL nerve catheter was comparable to that of autologous nerves (Reid et al., 2013). However, PCL conduit has high rigidity, which is inconvenient for clinical use. However, the degradation products of synthetic polymers such as PGA, PLA, and PGLA are acidic, and may stimulate inflammatory responses to surrounding tissues and hinder peripheral nerve repair and regeneration.

Other natural materials, including native fibrin (Du et al., 2017; Wang et al., 2018; Razavi et al., 2021), collagen (Saeki et al., 2018; Yao et al., 2018; He et al., 2021; Wang et al., 2021; Zheng et al., 2021), keratin (Apel et al., 2008; Lin et al., 2012; Pace et al., 2014), alginate (Lin et al., 2017; Rahmati et al., 2021; Abdelbasset et al., 2022), chitin (Bak et al., 2017; Lu C. F. et al., 2021; Yang et al., 2022b), chitosan (Li et al., 2018; Vishnoi et al., 2019), and silk fibroin (Carvalho et al., 2021; Kim et al., 2021; Zhao et al., 2020), as well as extracellular matrix (ECM) (Li T. et al., 2021; Kong et al., 2021; Kong et al., 2022), have shown great potential in treating long gap nerve defects. For instance, Wang et al. prepared a nerve catheter using chitosan/chitin to achieve excellent angiogenesis in nerve regeneration process for successfully repairing the 10 mm sciatic nerve defect in rats (Wang et al., 2016). These natural biomaterials such as chitosan and collagen have good biocompatibility, but the *in vivo* degradation time is too long to match the process of peripheral nerve regeneration. In addition, ECM has good biodegradability and biomimetic structure, but its application is mainly limited to the standardization of the decellular method and sterilization methods. To meet the various requirements for nerve regeneration, combination of different raw materials into one graft is reasonable to prepare nerve grafts with excellent chemical and physical performance.

### 3.2 Bionic structures

**FIGURE 1:**
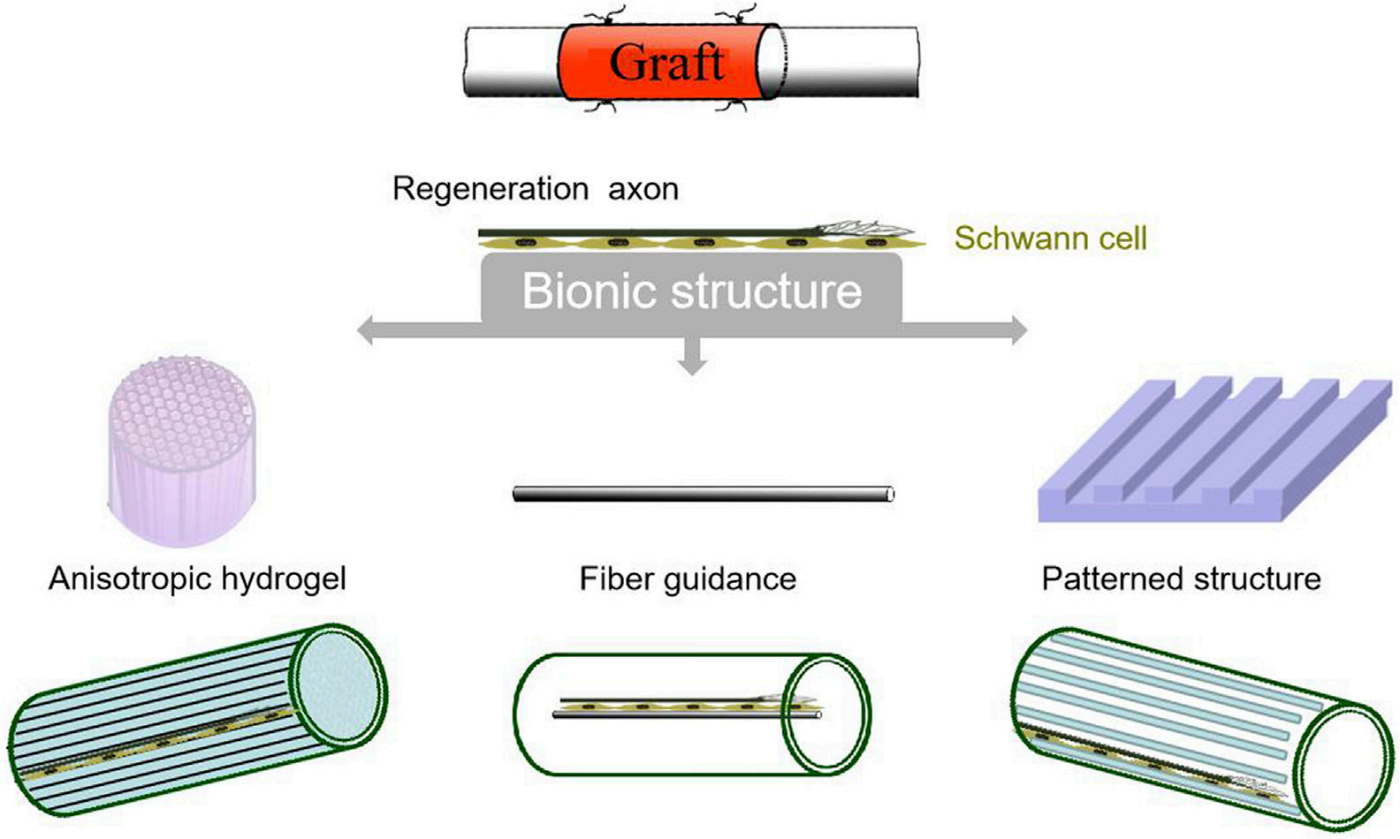
Strategies for constructing bionic structures of grafts.

It is well known that the peripheral nerve is morphologically composed of many root nerve fibers. The fibers form the root nerve bundles which then constitute the nerve stems (Tezcan, 2017). Neural fibers are aligned in parallel. Once the peripheral nerve is impaired, the distal nerve axon and myelin undergo fragmentation and collapse, leading to the invasion of a large number of phagocytes to clear axon myelin debris and more importantly, to stimulate the resting Schwann cells to divide and proliferate. After that, the stimulated cells keep growing along each nerve fiber intima and basement membrane into a line, named Buengner cell zone (Mirsky and Jessen, 1999). Eventually, the regenerated axons growing from the proximal end extend along the pipeline to the target organ, thereby restore its function. Therefore, the morphology of biomaterials should be beneficial to induce Schwan cells to arrange into lines and provide a pipeline for the extension of regenerated nerves (Figure 1). Bionic structures can provide a suitable microenvironment for the migration, proliferation and functionalization of nerve cells, and provides contact guidance for the directional growth of axons to improve the accuracy of nerve alignment. However, due to the complexity of natural neural structures, it still remains great challenges to prepare artificial nerve grafts with nerve-like structures in micro-nano scales. Current studies mainly focus on isotropic hydrogel fillers to provide intraluminal support for nerve regeneration (Huang et al., 2018), fibrotic intraluminal topographic guidance for neurites (Zou et al., 2016), and patterned cavity stand to provide three-dimensional (3D) structural support for nerve growth (Qu et al., 2011). For example, Du et al. prepared a 3D hierarchically arranged fibrin nanofiber hydrogel (AFG) to resemble the structure and biological function of natural fibrin cables. The AFG served as filler for tissue-engineered chitosan tubes, bridging a 10-mm-long sciatic nerve defect in rats. The results showed that AFG provided a suitable microenvironment that supported Schwann cell cable formation and axon regeneration within 2 weeks. (Du et al., 2017).

### 3.3 Improvement of regenerative microenvironment

Nerve injury regeneration is a very complex pathophysiological process and the local microenvironment has strong temporal and spatial characteristics, covering different levels such as molecules, cells, and organisms, and involving many fields such as physiology, pathology, biochemistry, biophysics, and bioinformatics (Rajaram et al., 2012; Chen et al., 2015; Radhakrishnan et al., 2015; Li G. et al., 2021). It was found that the use of artificial nerve grafts alone can only repair peripheral nerve defects in short distance. It is difficult to support the regeneration of large gap defects, which may be due to the lack of local support of cells and neurotrophic factors (Li T. et al., 2021; Chen et al., 2022). Nerve regrowth largely depends on the biological function exerted by the neurotrophins secreted by the nerve cells for regulating cell viability, migration, and differentiation in the peripheral and central nervous systems. Therefore, nerve conduits with neurotrophic factors or related components are essential tools for nerve repair (Gordon, 2009). For peripheral nerve defects with large gaps, insufficient fibrin formation between proximal and distal nerves restricts Schwann cell motility and Büngner band formation. Hence, the nutrient support of nerve conduits for cells is indispensable. Similar to skin tissue repair materials by encapsulation, graft copolymerization and so on, adding probiotics (Zhu et al., 2021), growth factors (Hao et al., 2022), etc., to improve the regenerative microenvironment, the functionalization of nerve repair materials can be achieved by similar means. Nerve conduits loaded with factors such as vascular endothelial growth factor (VEGF) (Rao et al., 2020), nerve growth factor (NGF) (Fine et al., 2002), insulin-like growth factor-I (IGF-I) (Zhang J. et al., 2022), fibroblast growth factor (FGF) (Ma et al., 2017), and glial growth factor (GGF) (Alsmadi et al., 2018), have been proven to have a positive role in peripheral nerve regrowth. (Kemp et al., 2008; Catrina et al., 2013). Yao et al. used longitudinally oriented collagen conduitloaded with NGF to repair 35-mm-long sciatic nerve defects in Beagle. After 9 months, the graft effectively achieved the sciatic nerve axonal regeneration and recovery of motor function than LOCC alone (Yao et al., 2018).

In addition, many types of physical or chemical stimulation, such as pH, optical signal, and electric current, can also promote nerve repair by activating the release of bioactive factors in neural cells (Tang et al., 2021a; Tang et al., 2021b; Chen et al., 2022; Tang et al., 2022). A growing amount of studies have found that electric stimulation can regulate Schwann cell proliferation (Gu et al., 2015b), neuronal differentiation (Meng et al., 2020), axon regrowth (Gordon, 2016), and the neurotrophic factors production (English et al., 2007). Some studies suggest that extracellular electrical signals can control gene expression, growth factor release, cell polarization and remodeling, etc. (Xue et al., 2018).

### 3.4 Seed cells

Seed cells are an essential component for tissue engineering, which can proliferate and differentiate to form target tissues to repair defects (Liu et al., 2022). Functional seed cells combined within nerve scaffolds can secrete growth factors, provide a favorable microenvironment for nerve regeneration, and further accelerate the nerve regeneration process. Ideal tissue-engineered neural seed cells require a wide range of sources, good safety, high efficacy without ethics and immune rejection (Lategan et al., 2022; Tang et al., 2022). Peripheral nerve tissue has a particular structure, and its regeneration includes regeneration of non-dead and functional neurons and myelin sheath. Highly differentiated neurons cannot be used as seed cells due to their inability of proliferation and division. On the other hand, glial cells and Schwann cells in peripheral nerve tissue playing important roles in regeneration process can proliferate and differentiate into mature cells to promote peripheral nerve regeneration combing with grafts (Ghane et al., 2021).

Neural stem cells can also differentiate into neurons and glia, the two major cell types of the peripheral and central nervous systems. As seed cells, they can promote the maturation of the nervous system. It has been shown that neural stem cells can promote the recovery of motor function by differentiation into locomotor neurons that innervate muscles. Neural stem cells also secrete a series of neurotrophic factors that promote the repair of nerve function. A novel stem cell-based pulsed electrical stimulation therapy was developed Du et al. for repairing 15 mm sciatic nerve injury of athymic nude mice. The results showed a significant improvement in locomotor function recovery and differentiation of Schwann cell using stem cells therapy, which was comparable to autograft in treatment of long gapped defects (Du et al., 2018). In addition, neural stem cells would promote angiogenesis and immune regulation (Wang et al., 2017). Moreover, BMSCs, olfactory sheath cells, etc., have also been used as seed cells for tissue-engineered nerves (Cooney et al., 2016; Sayad Fathi and Zaminy, 2017; Miah et al., 2021).

## 4 Prospects and challenges

Given the inherent drawbacks of autologous transplantation, the development of artificial nerve grafts is considered as a promising strategy in the field of peripheral nerve. Researchers in tissue engineering have focused on the development of biomaterials and tissue processing techniques to create various types of equipment and substrates to support peripheral nerve regeneration. In the past few decades, breakthroughs have been made in materials science and tissue engineering techniques to mimic or protect the neural tissue microenvironment for promoting nerve regrowth. Allogeneic nerve grafts and artificial catheters have been increasingly applied in clinical practice. At present, artificial and non-artificial nerve grafts have been achieved good clinical outcomes in treating short-distance peripheral nerve defects. However, there is still no commercial product that can be comparable to autologous transplantation in treating long gaps defects in large nerve. Effective control of growth factors release in the process of nerve regeneration in real-time still remains a challenge in this field. Notably, numerous studies are devoted to promote the directional growth of cells and remyelination by altering the morphology and structure within the grafts, or involving seed cells/growth factors to create better microenvironments for improving locomotor function recovery after nerve injury. However, most of the established neural stem cell lines come from mice, but there are obvious species differences between mice and humans. Therefore, the source, isolation, culture and identification of neural stem cells should be optimized, and the mechanism of neural stem cell differentiation needs to be further studied. In summary, These ongoing studies have laid a solid foundation for the development of future tissue engineering products for peripheral nerve repair.
